# Prevalence and risk factors of voice disorders among tour guides in Cappadocia

**DOI:** 10.1007/s00405-026-10328-3

**Published:** 2026-05-28

**Authors:** Namık Yücel Birol, Esra Yaşar Gündüz, Ferhat Alkan, Zübeyir Tutuş

**Affiliations:** 1 Department of Speech and Language Therapy, School of Health Sciences, Cappadocia University, Nevşehir, Türkiye; 2 Department of Speech and Language Therapy, Faculty of Health Sciences, Tarsus University, Mersin, Türkiye; 3 Department of Speech and Language Therapy, Institute of Graduate Education, İstinye University, İstanbul, Türkiye

**Keywords:** Tour guides, Voice disorders, Prevalence, Risk factors

## Abstract

**Purpose:**

This study aimed to determine the prevalence and risk factors of voice disorders among professional tour guides working in the Cappadocia region of Türkiye, a globally renowned tourism destination with high tourist density.

**Methods:**

A cross-sectional quantitative design was employed. Eighty-five licensed tour guides (41 females, 44 males; mean age = 42.9 ± 12.2 years) participated by completing an online self-report questionnaire developed for the Cappadocia context. The 61-item survey assessed demographics, vocal symptoms, phonotraumatic behaviors, occupational, lifestyle, and health-related risk factors. Descriptive statistics, chi-square tests, and logistic regression analyses were conducted using SPSS 27.

**Results:**

Voice problems were reported by 42.4% of guides during their careers (95% CI: 32.4–53.0) and 16.5% currently (95% CI: 10.1–25.8), while 23.5% experienced aphonia at least once annually (95% CI: 15.8–33.6). The most prevalent symptoms were throat dryness (77.6%), vocal fatigue (65.9%), and hoarseness (56.5%). Speaking loudly and continuing to talk despite voice problems were significantly associated with vocal complaints (*p* = .039 and *p* < .001, respectively). Environmental and health factors, including guiding noisy groups, long tour durations, and ear–nose–throat (ENT)-related conditions (sinus and other ENT disorders), increased the likelihood of voice problems. Logistic regression identified ENT disorders as an independent predictor (adjusted odds ratio [aOR] = 6.12, 95% CI: 1.33–28.17, *p* = .020).

**Conclusion:**

Voice disorders are common among Cappadocia tour guides and are associated with both occupational and health-related factors. These findings suggest that implementing voice hygiene education, ergonomic adaptations, and occupational health monitoring may help reduce risk and support vocal sustainability in this professional group.

**Supplementary Information:**

The online version contains supplementary material available at 10.1007/s00405-026-10328-3.

## Introduction

In a third of all occupations, workers rely on their voice as their primary tool [1]. Prior studies indicate that professional voice users face a higher risk of developing voice disorders due to the intensive and sustained use of their voice [2]. Voice disorders have a detrimental impact on professional voice users by lowering their quality of life and raising healthcare costs that burden society [3, 4]. Awareness of the relationship between voice disorders and occupational factors has increased, and these conditions are now recognized as occupational disorders [5]. Roy et al. (2004) provided a comprehensive self-reported definition of voice disorder as any occasion when the voice fails to function, perform, or sound as it normally does, leading to communication difficulties, particularly when speaking is a professional necessity [6].

Occupational voice disorders arise from the intensive use of the voice while performing professional duties. These disorders may develop not only due to vocal overuse but also as a result of various environmental and individual factors. These factors include working conditions, personal voice care habits, stress levels, acoustic environment, and ergonomic factors. Affected individuals typically include those who rely on their voice as the primary tool of their profession, such as teachers, performing artists, call center workers, clergy (e.g., priests), and healthcare professionals [7].

Tour guides fall within the category of occupational voice users, as their work requires frequent and prolonged speaking, often in challenging acoustic or environmental conditions. These challenges include speaking in noisy museums, echoic buildings with high ceilings, open-air tours affected by weather, or quiet venues such as art galleries. In these venues, tour guides must consciously increase their vocal intensity, sometimes without the aid of microphones, which may not be practical in every setting [8, 9]. As part of their profession, tour guides accompany travelers and introduce them to sites of interest [10]. A large-scale study conducted in France demonstrated that voice disorders were highly prevalent among tour guides [6, 11]. In particular, it was reported that 21.3% of the tour guides presented with clinical voice problems and nearly 45% reported at least one episode of aphonia annually, markedly higher than the 6.2% prevalence observed in the general population. Key risk factors identified include prolonged loud voice use, exposure to noise and weather variations, stress, back pain and lack of professional training in voice care [11]. In another study conducted in Finland, it was found that 11% of guides experienced four or more frequently occurring vocal symptoms that met the criteria for a voice disorder [9]. The most commonly reported symptom in this study was the need for throat clearing during speech, affecting 13.7% of participants. In addition, the study identified several health-related risk factors such as asthma, chronic rhinitis, psychological stress, and the frequency of guiding. These risk factors were significantly associated with an increased number of vocal symptoms. Environmental factors were also prominent in that study: 30.3% of the guides reported experiencing reverberation, and 31.5% reported disturbing background noise on a weekly basis or more [9]. While the prevalence in this sample was somewhat lower than in other vocally demanding occupations, the authors suggested that this may be due to factors such as lower guiding frequency, better vocal technique, or methodological differences in how disorders were defined.

Cappadocia, located in Türkiye, is a globally renowned tourism destination characterized by its historical heritage, unique geological formations, and rich cultural landscape [12]. The region, listed as a UNESCO World Heritage Site, attracts millions of domestic and international visitors each year with its fairy chimneys, underground cities, and historic churches. In fact, Cappadocia has been visited by approximately 2.5 million tourists annually in recent years [13]. This intense visitor flow indicates that tourism activities in the region operate continuously and at a demanding pace. This, in turn, increases the physical and occupational workload of professionals, particularly tour guides.

In addition to voice disorders, tour guides are also vulnerable to various other occupational health problems because of the physical and psychological demands of the profession. A recent mixed-methods study conducted in Türkiye reported that many tour guides experience musculoskeletal pain caused by prolonged walking, long-standing hours, and physically demanding work environments [14]. The same study also revealed that stress and burnout are common among guides, particularly because of long working hours, irregular schedules, and the pressure of meeting both tourist expectations and employer demands. Similarly, Ulusoy Mutlu et al. (2024) found that musculoskeletal disorders and psychological strain were the most prevalent health issues reported by tour guides [15]. These findings highlight the challenges faced by tour guides and emphasize the need for comprehensive preventive strategies that address both physical and mental wellbeing.

Although most risk factors for voice disorders among occupational voice users have been identified, their exact significance for tour guides is not fully understood. Given the unique environmental, cultural, and occupational characteristics of the Cappadocia region, it is essential to assess the prevalence and variety of risk factors of voice disorders specifically among tour guides working in this region. Since no published study has investigated voice disorders in this population, we aimed to examine the prevalence and related risk factors of voice disorders among tour guides working in Cappadocia. We believe that this study is particularly important for increasing awareness of vocal health within this professional group and for developing preventive strategies. The following research questions (RQs) were addressed in the present study:

- RQ-1: What is the prevalence of voice disorders among tour guides working in the Cappadocia region?
- RQ-2: What are the risk factors associated with voice disorders among tour guides working in the Cappadocia region?

## Methods

### Study design

This research was designed as a quantitative study with a cross-sectional design.

A questionnaire was used to examine the prevalence of voice problems among tour guides working in the Cappadocia region of Türkiye and to identify potential risk factors that may contribute to these problems. All eligible tour guides registered with the Nevşehir Chamber of Tour Guides (NERO) were invited to participate through an open invitation approach covering the entire sampling frame. The final sample was formed via voluntary response, consistent with a convenience sampling procedure. The Nevşehir Chamber of Tour Guides (NERO) is the local professional chamber for licensed tour guides in the Cappadocia region and provided the sampling frame and recruitment channels for this study. Outcomes, including self-reported current and career voice problems and related symptoms, and potential risk factors, including phonotraumatic behaviors as well as occupational, lifestyle, and health-related factors, were assessed concurrently using a standardized, self-administered online questionnaire.

### Participants

The study population consisted of a total of 472 tour guides actively working in the Cappadocia region of Türkiye and registered with NERO [16]. The sample size was determined using the Raosoft online sample size calculator, based on parameters of a 10% margin of error, a 95% confidence level, and a 50% response distribution. According to these criteria, the minimum required sample size was calculated as 80 participants.

**Fig. 1:**
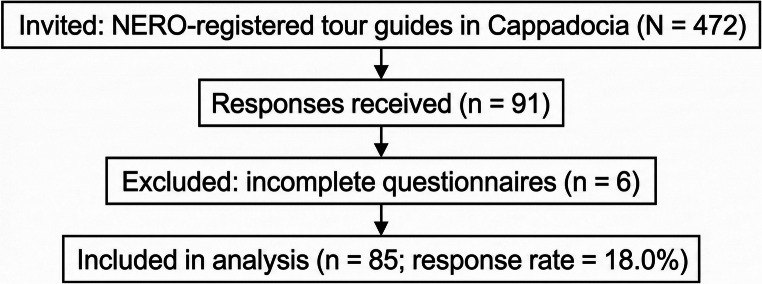
Study flow diagram

All eligible NERO members were invited to participate via WhatsApp groups and e-mail lists, and a QR (quick response) code was also displayed during a face-to-face seminar organized by NERO. The study flow is summarized in Fig. 1. A total of 91 responses were received; six questionnaires were not completed and were excluded, yielding a final analytic sample of 85 participants (response rate: 18.0%). Inclusion criteria were age ≥ 18 years, actively working in the Cappadocia region for at least six months, and holding a valid professional license (badge). The absence of a valid license and incomplete questionnaires were determined as the exclusion criteria.

### Questionnaire on the prevalence of voice disorders and related risk factors among tour guides

A self-reported questionnaire (see Supplementary Material) was developed by the researchers to determine the prevalence of voice disorders and related risk factors among tour guides in the Cappadocia region. Consistent with approaches used in prior occupational voice questionnaires, our questionnaire was primarily developed based on the instruments used by Sanssené et al. (2020) and Cansu (2020) [9, 11]. However, modifications were implemented to account for environmental factors specific to the Cappadocia region, as well as for certain cultural and linguistic adaptations. To support face and content validity, the draft questionnaire was reviewed by an expert panel consisting of three lecturers from the Department of Tour Guiding at Cappadocia University and three speech-language pathologist (SLP) academics with expertise in voice. Panel feedback focused on item relevance, comprehensiveness, clarity, and feasibility for tour guides; minor revisions were made accordingly.

The finalized questionnaire consisted of seven sections and 61 items: demographic information, prevalence of voice problems, symptoms, phonotraumatic behaviors, and risk factors (occupational, lifestyle, and health-related). The demographic section included descriptive items such as age, gender, and length of professional experience. The second section included questions about the prevalence of voice problems, such as current (point prevalence) voice problems and frequency of aphonia, as self-reported by the participants. The third section addressed symptoms such as hoarseness, throat pain, and vocal fatigue. The remaining four sections focused on risk factors associated with voice problems. The phonotraumatic behaviors section included items such as speaking loudly, excessive talking, and frequent throat clearing. The occupational risk factors section covered aspects such as duration of loud speaking and the average length of a single tour. Lifestyle-related risk factors comprised items such as consumption of spicy and fatty foods, smoking, sleep duration, and beverage intake (coffee, tea, cola/energy drinks, water, and alcohol). Health-related risk factors focused on items concerning medical conditions that could affect the voice, such as asthma, sinus problems, and nasal allergies.

The questionnaire included a variety of response formats such as dichotomous (e.g., Yes/No), Likert-type scales (e.g., Never–Rarely–Sometimes–Often–Always), ordinal frequency ranges (e.g., 1–5 h), and multiple-choice questions allowing for more than one response. This structure enabled the collection of both categorical and ordinal data suited for comprehensive descriptive and inferential statistical analyses. Outcome measures were anchored to specific reference periods to improve interpretability: current voice problems referred to the participant’s status at the time of survey completion, career voice problems referred to whether a problem had occurred at any point since starting work as a tour guide, and aphonia frequency was captured using ordinal categories reflecting a typical year (never; once or twice per year; three or more times per year). Occupational exposure items were similarly time-referenced (e.g., a typical week during tours, a typical tour, and typical high-season duration). These time-anchored outcome definitions and the single-administration survey approach are consistent with prior survey-based studies of occupational/professional voice users [17–19].

### Data collection process

The questionnaire was hosted online via Microsoft Forms and shared through the WhatsApp groups and e-mail lists of active members of NERO. Participants also completed the questionnaire by scanning a QR code displayed on the screen during a face-to-face seminar organized by NERO for tour guides. The questionnaire was self-administered in a uniform format with an identical item order and response options for all participants, and no researcher was present during completion. This helped to minimize observer bias. Responses were anonymous; no names or IP addresses were collected. To minimize duplicate entries, the form was configured to allow one response per account, and mandatory fields were used to reduce missing data. The average completion time of the instrument was approximately 10 min. Data were collected between November 5 and 25, 2024, during the high season, when tourists are most active in Cappadocia [20]. This time window was selected to capture tour guides during peak occupational vocal demand and to reduce potential seasonal variation in exposure. Participants were provided with detailed information about the study and were included only after providing informed consent. Ethical approval for the study was obtained from the Non-Interventional Clinical Research Ethics Committee of Cappadocia University on October 6, 2024 (File No: E-64577500-050.99-91324; Decision No: 24.18 G.O.), indicating that the study met al.l ethical standards and posed no ethical concerns.

### Data analysis

Statistical analyses were conducted using IBM SPSS Statistics version 27.0. Variables were summarized using descriptive statistics, with categorical variables presented as frequencies and percentages. Continuous variables, when applicable, were summarized as means and standard deviations; however, inferential analyses in this study were primarily based on categorical comparisons, as the key outcome and most predictors were categorical or categorized for analysis. In line with previous literature, participants were divided into two groups: those who reported experiencing voice problems at any point after starting their careers and those who did not experience voice problems throughout their careers [17–19]. Associations between categorical variables were evaluated using Pearson’s chi-square test when expected cell counts were adequate. When expected cell frequencies were low (particularly in 2 × 2 tables), Fisher’s exact test was applied; for larger contingency tables with sparse cells (e.g., 2 × 3), the Fisher–Freeman–Halton test was used. To facilitate interpretation beyond statistical significance, effect sizes for contingency analyses were reported as Phi (2 × 2) or Cramer’s V for larger contingency tables (e.g., 2 × 3, 2 × 4). Given the number of bivariate comparisons, these analyses were interpreted as exploratory; therefore, p-values were considered cautiously and interpreted alongside effect sizes, while primary inference was based on the multivariable model. Prior to fitting the multivariable logistic regression model, potential collinearity among candidate predictors was assessed, and no evidence of problematic collinearity was identified. To examine associations between the presence of career voice problems and potential risk factors, multiple logistic regression was performed using forward stepwise (Wald) selection, and unadjusted and adjusted odds ratios (aOR) with 95% confidence intervals (CIs) were reported. In addition, 95% confidence intervals were calculated for the main prevalence estimates. The level of statistical significance was set at *p* < .05 for all analyses.

## Results

### Prevalence of voice problems

A total of 85 professional tour guides (41 females, 44 males) participated in the study, with a mean age of 42.86 years (SD = 12.20; range: 24–67 years). Among the participants, 16.5% (*n* = 14; 95% CI: 10.1–25.8) reported currently experiencing voice problems (point prevalence), while 42.4% (*n* = 36; 95% CI: 32.4–53.0) stated that they had experienced voice problems at some point during their professional career (career prevalence). In addition, 23.5% of all participants (*n* = 20; 95% CI: 15.8–33.6) reported experiencing complete voice loss (aphonia) at least once during their professional practice. Among these individuals, 85% (*n* = 17) indicated that they experienced aphonia once or twice per year, while 15% (*n* = 3) reported three or more annual episodes of complete voice loss.

In accordance with previous literature [17–19], participants were divided into two groups based on the presence or absence of career-related voice problems. All subsequent comparisons were made between guides who reported having experienced voice problems at any point in their career (career VP group; *n* = 36) and those who did not (no VP group; *n* = 49).

### Demographic characteristics of tour guides

The demographic characteristics of the participants are summarized in Table 1. No statistically significant differences were observed between the career VP group (*n* = 36) and the no VP group (*n* = 49) regarding age (χ² = 2.802, *p* = .246), gender distribution (χ² = 0.026, *p* = .873), educational level (χ² = 0.387, *p* = .534), years of professional experience (χ² = 3.050, *p* = .218), having additional job requiring prolonged voice use (χ² = 0.012, *p* = .913), or engaging in a hobby involving voice use (χ² = 0.041, *p* = .839). These results suggest that demographic and background characteristics were comparable across groups.

**Table 1:** Participant characteristics by career voice-problem status

Features		Career VP Group (*n* = 36) *N* (%)	No VP Group (*n* = 49) *N* (%)	χ2	df	*p*	Effect size (Cramer’s V/Φ)
Age	18–30	6 (16.7)	14 (28.6)	2.802	2	0.246	0.182
	31–50	21 (58.3)	20 (40.8)				
	Over 50	9 (25)	15 (30.6)				
Gender	Female	17 (47.2)	24 (49)	0.026	1	0.873	0.017
	Male	19 (52.8)	25 (51)				
Education level	Undergraduate	25 (69.4)	37 (75.5)	0.387	1	0.534	0.067
	Graduate	11 (30.6)	12 (24.5)				
Years of professional experience	0–4	9 (25)	21 (42.9)	3.050	2	0.218	0.189
	5–10	8 (22.2)	7 (14.3)				
	More than 10	19 (52.8)	21 (42.9)				
Additional job requiring prolonged voice use	Yes	7 (19.4)	10 (20.4)	0.012	1	0.913	0.012
	No	29 (80.6)	39 (79.6)				
Hobby involving voice use	Yes	6 (16.7)	9 (18.4)	0.041	1	0.839	0.022
	No	30 (83.3)	40 (81.6)				

* p < .05 was considered statistically significant. Career VP indicates participants who reported experiencing voice problems at any point after starting their careers. p values are from Pearson chi-square unless Fisher’s Exact/Fisher–Freeman–Halton tests were used when expected cell counts were low. Abbreviations: *VP* voice problems, *χ2* Pearson chi-square statistic, *df* degrees of freedom, *Φ* phi coefficient for 2 × 2 tables, *V* Cramer’s V for larger contingency tables.

### Vocal symptoms experiences by tour guides

As shown in Table 2, the career VP group reported a significantly higher frequency of various vocal symptoms compared to those without such problems. The most prevalent symptoms among all participants, regardless of group, were throat dryness (77.6%, *n* = 66), vocal fatigue (65.9%, *n* = 56), throat tightness (62.4%, *n* = 53), throat discomfort (62.4%, *n* = 53), hoarseness (56.5%, *n* = 48), and difficulty speaking loudly (56.5%, *n* = 48).

**Table 2:** Vocal symptoms experienced by tour guides during or after guiding

Features		Career VP Group (*n* = 36) *N* (%)	No VP Group (*n* = 49) *N* (%)	χ2	df	*p*	Effect size (Cramer’s V/Φ)
Hoarseness	Yes	35 (97.2)	13 (26.5)	42.190	1	< 0.001*	0.705
	No	1 (2.8)	36 (73.5)				
Throat dryness	Yes	32 (88.9)	34 (69.4)	4.547	1	0.033*	0.231
	No	4 (11.1)	15 (30.6)				
Vocal fatigue	Yes	32 (88.9)	24 (49)	14.705	1	< 0.001*	0.416
	No	4 (11.1)	25 (51)				
Throat tightness	Yes	32 (88.9)	21 (42.9)	18.733	1	< 0.001*	0.469
	No	4 (11.1)	28 (57.1)				
Sudden loss of voice	Yes	24 (66.7)	13 (26.5)	13.600	1	< 0.001*	0.400
	No	12 (33.3)	36 (73.5)				
Throat discomfort	Yes	30 (83.3)	23 (46.9)	11.710	1	< 0.001*	0.371
	No	6 (16.7)	26 (53.1)				
Shortness of breath	Yes	14 (38.9)	13 (26.5)	1.462	1	0.227	0.131
	No	22 (61.1)	36 (73.5)				
Difficulty speaking loudly	Yes	32 (88.9)	16 (32.7)	26.699	1	< 0.001*	0.560
	No	4 (11.1)	33 (67.3)				
Throat pain	Yes	27 (75)	17 (34.7)	13.503	1	< 0.001*	0.399
	No	9 (25)	32 (65.3)				
Complete voice loss	Yes	10 (27.8)	3 (6.1)	7.512	1	0.006*	0.297
	No	26 (72.2)	46 (93.9)				

* p < .05 was considered statistically significant. Career VP indicates participants who reported experiencing voice problems at any point after starting their careers. p values are from Pearson chi-square unless Fisher’s Exact/Fisher–Freeman–Halton tests were used when expected cell counts were low. Abbreviations: *VP* voice problems, *χ2* Pearson chi-square statistic, *df* degrees of freedom, *Φ* phi coefficient for 2 × 2 tables, *V* Cramer’s V for larger contingency tables.

When comparing the groups, those in the career VP group reported much higher rates of these symptoms. Hoarseness was reported by 97.2% of guides in the career VP group compared to 26.5% in the no VP group (*p* < .001, Φ = 0.705). Similarly, vocal fatigue (88.9% vs. 49.0%; *p* < .001, Φ = 0.416), throat dryness (88.9% vs. 69.4%; *p* = .033, Φ = 0.231), and throat tightness (88.9% vs. 42.9%; *p* < .001, Φ = 0.469) were each reported significantly more often in the career VP group. Difficulty speaking loudly (88.9% vs. 32.7%; *p* < .001, Φ = 0.560), throat discomfort (83.3% vs. 46.9%; *p* < .001, Φ = 0.371), throat pain (75.0% vs. 34.7%; *p* < .001, Φ = 0.399), sudden loss of voice (66.7% vs. 26.5%; *p* < .001, Φ = 0.400), and complete voice loss (27.8% vs. 6.1%; *p* = .006, Φ = 0.297) also showed significant between-group differences. The only symptom that did not differ significantly between groups was shortness of breath (χ² = 1.462, *p* = .227). These findings indicate a strong relationship between career voice problems and the experience of multiple vocal symptoms among tour guides.

### Phonotraumatic behaviors exhibited by tour guides

As presented in Table 3, phonotraumatic behaviors were highly prevalent among tour guides for both groups. Regardless of voice problem status, 88.2% (*n* = 75) of the participants reported speaking loudly, 82.4% (*n* = 70) excessive talking, and 65.9% (*n* = 56) speaking while having a voice problem.

**Table 3:** Phonotraumatic behaviors of tour guides with and without voice problems

		Career VP Group (*n* = 36) *N* (%)	No VP Group (*n* = 49) *N* (%)	χ2	df	*p*	Effect size (Cramer’s V/Φ)
Speaking loudly	Yes	35 (97.2)	40 (81.6)	4.859	1	0.039*	0.239
	No	1 (2.8)	9 (18.4)				
Excessive talking	Yes	32 (88.9)	38 (77.6)	1.836	1	0.175	0.147
	No	4 (11.1)	11 (22.4)				
Speaking at an excessively fast rate	Yes	15 (41.7)	23 (46.9)	0.233	1	0.629	0.052
	No	21 (58.3)	26 (53.1)				
Frequent coughing	Yes	16 (44.4)	15 (30.6)	1.714	1	0.191	0.142
	No	20 (55.6)	34 (69.4)				
Frequent throat clearing	Yes	19 (52.8)	22 (44.9)	0.516	1	0.473	0.078
	No	17 (47.2)	27 (55.1)				
Holding breath while speaking	Yes	15 (41.7)	11 (22.4)	3.610	1	0.057	0.206
	No	21 (58.3)	38 (77.6)				
Speaking during a throat infection	Yes	25 (69.4)	27 (55.1)	1.797	1	0.180	0.145
	No	11 (30.6)	22 (44.9)				
Speaking while having a voice problem	Yes	31 (86.1)	25 (51)	11.369	1	< 0.001*	0.366
	No	5 (13.9)	24 (49)				

* p < .05 was considered statistically significant. Career VP indicates participants who reported experiencing voice problems at any point after starting their careers. p values are from Pearson chi-square unless Fisher’s Exact/Fisher–Freeman–Halton tests were used when expected cell counts were low. Abbreviations: *VP* voice problems, *χ2* Pearson chi-square statistic, *df* degrees of freedom, *Φ* phi coefficient for 2 × 2 tables, *V* Cramer’s V for larger contingency tables.

When comparing guides with and without career voice problems, several behaviors were more frequently observed in the career VP group. Specifically, speaking loudly was significantly more common in the career VP group (97.2%) than the no VP group (81.6%) (χ² = 4.859, *p* = .039, Φ = 0.239). Similarly, speaking while having a voice problem was reported by 86.1% of the career VP group, compared to 51.0% in the no VP group, yielding a statistically significant difference (χ² = 11.369, *p* < .001, Φ = 0.366).

### Occupation-related factors

As shown in Table 4, most occupational and environmental factors did not significantly differ between the career VP group and the no VP group. However, two variables emerged as significant risk factors. First, guiding tours in noisy group settings was reported by 61.1% of the career VP group compared to 38.8% of the no VP group, indicating a statistically significant difference (χ² = 4.146, *p* = .042, Φ = 0.221). Second, longer guiding duration per tour was also associated with voice problems: 86.1% of the career VP group reported conducting tours lasting 6 h or more, compared to 67.3% in the no VP group (χ² = 3.928, *p* = .047, Φ = 0.215).

**Table 4:** Work-related factors of tour guides with and without voice problems

Features		Career VP Group (*n* = 36) *N* (%)	No VP Group (*n* = 49) *N* (%)	χ2	df	*p*	Effect size (Cramer’s V/Φ)
Work Characteristics							
Average group size during high season	1–10	5 (13.9)	9 (18.4)	2.439	2	0.295	0.169
	11–30	17 (47.2)	15 (30.6)				
	> 31 people	14 (38.9)	25 (51)				
Guidance in the outdoor settings	Yes	35 (97.2)	49 (100)	1.377	1	0.424	0.127
	No	1 (2.8)	0 (0)				
Guidance in the indoor settings	Yes	16 (44.4)	19 (38.8)	0.275	1	0.600	0.057
	No	20 (55.6)	30 (61.2)				
Poor acoustics environment	Yes	14 (38.9)	15 (30.6)	0.632	1	0.426	0.086
	No	22 (61.1)	34 (69.4)				
Background noise	Yes	30 (83.3)	42 (85.7)	0.091	1	0.763	0.033
	No	6 (16.7)	7 (14.3)				
Dispersed group	Yes	26 (72.2)	37 (75.5)	0.117	1	0.732	0.037
	No	10 (27.8)	12 (24.5)				
Noisy group	Yes	22 (61.1)	19 (38.8)	4.146	1	0.042*	0.221
	No	14 (38.9)	30 (61.3)				
Voice use per week (during tours)	2–10 h	7 (19.4)	17 (34.7)	4.026	2	0.134	0.218
	11–20 h	5 (13.9)	10 (20.4)				
	> 21 h	24 (66.7)	22 (44.9)				
Weekly loud speaking time (during tours)	< 5 h	5 (13.9)	9 (18.4)	0.371	3	0.946	0.066
	6–15 h	13 (36.1)	18 (36.7)				
	16–20 h	5 (13.9)	6 (12.2)				
	> 21 h	13 (36.1)	16 (32.7)				
Guiding duration per tour	< 5 h	5 (13.9)	16 (32.7)	3.928	1	0.047*	0.215
	> 6 h	31 (86.1)	33 (67.3)				
Duration of high season (months)	1–6	21 (58.3)	34 (69.4)	1.110	1	0.292	0.114
	7–12	15 (41.7)	15 (30.6)				
Use of voice amplification device during tours	Never	5 (18.8)	11 (22.4)	4.407	4	0.354	0.228
	Rarely	10 (21.2)	8 (16.3)				
	Sometimes	14 (36.5)	17 (34.7)				
	Often	7 (20)	10 (20.4)				
	Always	0 (0)	3 (6.1)				
Tour Locations							
Museums (indoor)	Yes	32 (88.9)	43 (87.8)	0.026	1	0.873	0.017
	No	4 (11.1)	6 (12.2)				
Museums (outdoor)	Yes	35 (97.2)	42 (85.7)	3.224	1	0.073	0.195
	No	1 (2.8)	7 (14.3)				
Underground cities and cellars	Yes	30 (83.3)	37 (75.5)	0.761	1	0.383	0.095
	No	6 (16.7)	12 (24.5)				
Valleys	Yes	25 (69.4)	36 (73.5)	0.166	1	0.684	0.044
	No	11 (30.6)	13 (26.5)				
Fairy chimneys	Yes	26 (72.2)	35 (71.4)	0.006	1	0.936	0.008
	No	10 (27.8)	14 (28.6)				
Tour buses	Yes	34 (94.4)	42 (85.7)	1.671	1	0.196	0.140
	No	2 (5.6)	7 (14.3)				
City tours	Yes	30 (83.3)	41 (83.7)	0.002	1	0.967	0.005
	No	6 (16.7)	8 (16.3)				
Historical buildings and structures	Yes	30 (83.3)	42 (85.7)	0.091	1	0.763	0.033
	No	6 (16.7)	7 (14.3)				
Churches and mosques	Yes	33 (91.7)	46 (93.9)	0.155	1	0.694	0.043
	No	3 (8.3)	3 (6.1)				
Environmental Conditions							
Temperature change	Rarely	1 (2.8)	4 (8.2)	3.410	3	0.344	0.200
	Sometimes	13 (36.1)	16 (32.7)				
	Often	13 (36.1)	23 (46.9)				
	Always	9 (25)	6 (12.2)				
Windy weather	Never	0 (0)	1 (2)	4.352	4	0.307	0.226
	Rarely	1 (2.8)	6 (12.2)				
	Sometimes	28 (77.8)	30 (61.2)				
	Often	7 (19.4)	11 (22.4)				
	Always	0 (0)	1 (2)				
Humid weather	Never	1 (2.8)	3 (6.1)	3.476	4	0.480	0.202
	Rarely	6 (16.7)	10 (20.4)				
	Sometimes	25 (69.4)	25 (51)				
	Often	4 (11.1)	10 (20.4)				
	Always	0 (0)	1 (2)				
Dusty weather	Never	0 (0)	1 (2)	2.816	4	0.600	0.182
	Rarely	1 (2.8)	4 (8.2)				
	Sometimes	21 (58.3)	27 (55.1)				
	Often	10 (27.8)	9 (18.4)				
	Always	4 (11.1)	8 (16.3)				
Dry weather	Never	0 (0)	2 (4.1)	2.485	4	0.712	0.171
	Rarely	1 (2.8)	3 (6.1)				
	Sometimes	20 (55.6)	28 (57.1)				
	Often	11 (30.6)	10 (20.4)				
	Always	4 (11.1)	6 (12.2)				
Echo	Never	1 (2.8)	6 (12.2)	4.066	3	0.234	0.219
	Rarely	9 (25)	17 (34.7)				
	Sometimes	24 (66.7)	24 (49)				
	Often	2 (5.6)	2 (4.1)				
Stress and anxiety	Never	0 (0)	1 (2)	4.868	4	0.271	0.239
	Rarely	2 (5.6)	6 (12.2)				
	Sometimes	16 (44.4)	17 (34.7)				
	Often	12 (33.3)	22 (44.9)				
	Always	6 (16.7)	3 (6.1)				

* p < .05 was considered statistically significant. Career VP indicates participants who reported experiencing voice problems at any point after starting their careers. p values are from Pearson chi-square unless Fisher’s Exact/Fisher–Freeman–Halton tests were used when expected cell counts were low. Abbreviations: *VP* voice problems, *χ2* Pearson chi-square statistic, *df* degrees of freedom, *Φ* phi coefficient for 2 × 2 tables, *V* Cramer’s V for larger contingency tables.

While not statistically significant, a higher proportion of the career VP group also reported working in environments with poor acoustics, frequent exposure to dry or dusty weather, and elevated vocal demands during the high season. Furthermore, more than two-thirds of both groups reported guiding in open-air locations, underground cities, valleys, and historical sites. The use of a voice amplification device during tours did not significantly differ between the groups (χ² = 4.407, *p* = .354), and most guides indicated only occasional or rare use of such tools. Similarly, the groups did not significantly differ in terms of reported stress and anxiety levels during guiding.

### Lifestyle-related factors

As shown in Table 5, lifestyle-related factors were assessed in relation to the presence of career voice problems among tour guides. None of the factors examined reached statistical significance between the two groups (*p* > .05). However, certain trends were observed that may have potential clinical relevance.

**Table 5:** Lifestyle factors of tour guides with and without voice problems

Features		Career VP Group (*n* = 36) *N* (%)	No VP Group (*n* = 49) *N* (%)	χ2	df	*p*	Effect size (Cramer’s V/Φ)
Consuming spicy or fatty foods	Yes	26 (72.2)	38 (77.6)	0.317	1	0.574	0.061
	No	10 (27.8)	11 (22.4)				
Consuming very hot or very cold foods/drinks	Yes	23 (63.9)	28 (57.1)	0.394	1	0.530	0.068
	No	13 (36.1)	21 (42.9)				
Irregular meal timing	Yes	17 (47.2)	23 (46.9)	0.001	1	0.979	0.003
	No	19 (52.8)	26 (53.1)				
Smoking	Yes	9 (25)	18 (36.7)	1.318	1	0.251	0.125
	No	27 (75)	31 (63.3)				
Regular sleep	Yes	21 (58.3)	37 (75.5)	2.825	1	0.093	0.182
	No	15 (41.7)	12 (24.5)				
Sleep duration	6 h or less	14 (38.9)	17 (34.7)	0.158	1	0.691	0.043
	7 h or more	22 (61.1)	34 (65.3)				
Daily coffee consumption	2 cups or less	26 (72.2)	32 (65.3)	0.458	1	0.499	0.073
	More than 2 cups	10 (27.8)	17 (34.7)				
Daily energy drink/ cola consumption	2 cups or less	33 (91.7)	48 (98)	1.832	1	0.307	0.147
	More than 2 cups	3 (8.3)	1 (2)				
Daily tea consumption	2 cups or less	12 (33.3)	19 (38.8)	0.265	1	0.607	0.056
	More than 2 cups	24 (66.7)	30 (61.2)				
Daily alcohol intake	None	25 (69.4)	31 (63.3)	0.886	2	0.675	0.102
	2 cups or less	8 (22.2)	15 (30.6)				
	More than 2 cups	3 (8.3)	3 (6.1)				
Daily water consumption	8 cups or less	23 (63.9)	29 (59.2)	0.193	1	0.822	0.048
	More than 8 cups	13 (36.1)	20 (40.8)				
Daily physical activity	Less than 30 min	6 (16.7)	11 (22.4)	0.434	1	0.510	0.071
	More than 30 min	30 (83.3)	38 (77.6)				

* p < .05 was considered statistically significant. Career VP indicates participants who reported experiencing voice problems at any point after starting their careers. p values are from Pearson chi-square unless Fisher’s Exact/Fisher–Freeman–Halton tests were used when expected cell counts were low. Abbreviations: *VP* voice problems, *χ2* Pearson chi-square statistic, *df* degrees of freedom, *Φ* phi coefficient for 2 × 2 tables, *V* Cramer’s V for larger contingency tables.

The consumption of spicy or fatty foods was reported by 72.2% of the career VP group and 77.6% of the no VP group. Similarly, the consumption of very hot or cold foods/drinks was common in both groups (63.9% vs. 57.1%). Irregular meal timing was reported by nearly half of the participants in each group (47.2% vs. 46.9%). Sleep-related factors also did not show significant group differences. While 58.3% of the career VP group reported regular sleep patterns, this rate was higher (75.5%) among the no VP group (χ² = 2.825, *p* = .093). Both groups had similar proportions of participants who slept less than 6 h per night. Regarding beverage consumption, over 60% of both groups drank more than two cups of tea daily, and more than 90% consumed two or fewer cups of energy drinks or cola. Alcohol intake, daily water consumption, and physical activity levels were also comparable between the groups. Approximately 64% of the total sample reported drinking less than 8 cups of water per day, and the majority in both groups reported engaging in more than 30 min of daily physical activity.

In summary, although no statistically significant differences were found, several lifestyle patterns, such as suboptimal hydration, inconsistent sleep, and frequent consumption of potentially irritating foods or beverages, were commonly observed.

### Health-related factors

As shown in Table 6, two health-related factors were significantly associated with career voice problems among tour guides. Sinus problems were reported by 50.0% of the career VP group compared to only 20.4% of the no VP group (χ² = 8.227, *p* = .004, Φ = 0.311). Additionally, other ear, nose, and throat (ENT) disorders were reported by 33.3% of the career VP group, compared to only 6.1% in the no VP group, demonstrating a highly significant association (χ² = 10.573, *p* = .001, Φ = 0.353).

**Table 6:** Health-related factors of tour guides with and without voice problems

		Career VP Group (*n* = 36) *N* (%)	No VP Group (*n* = 49) *N* (%)	χ2	df	*p*	Effect size (Cramer’s V/Φ)
Asthma	Yes	4 (11.1)	1 (2)	3.084	1	0.158	0.190
	No	32 (88.9)	48 (98)				
Sinus problems	Yes	18 (50)	10 (20.4)	8.227	1	0.004*	0.311
	No	18 (50)	39 (79.6)				
Nasal allergies	Yes	13 (36.1)	12 (24.5)	1.350	1	0.245	0.126
	No	23 (63.9)	37 (75.5)				
Frequent colds	Yes	12 (33.3)	11 (22.4)	1.246	1	0.264	0.121
	No	24 (66.7)	38 (77.6)				
Difficulty in hearing normal conversations	Yes	3 (8.3)	5 (10.2)	0.085	1	1.000	0.032
	No	33 (91.7)	44 (89.8)				
Acid reflux or heartburn	Yes	13 (36.1)	16 (32.7)	0.110	1	0.740	0.036
	No	23 (63.9)	33 (67.3)				
Neurological disorders	Yes	2 (5.6)	4 (8.2)	0.215	1	1.000	0.050
	No	34 (94.4)	45 (91.8)				
Regular medication use	Yes	9 (25)	11 (22.4)	0.075	1	0.784	0.030
	No	27 (75)	38 (77.6)				
Other ear, nose, and throat disorders	Yes	12 (33.3)	3 (6.1)	10.573	1	0.001*	0.353
	No	24 (66.7)	46 (93.9)				

* p < .05 was considered statistically significant. Career VP indicates participants who reported experiencing voice problems at any point after starting their careers. p values are from Pearson chi-square unless Fisher’s Exact/Fisher–Freeman–Halton tests were used when expected cell counts were low. Abbreviations: *VP* voice problems, *χ2* Pearson chi-square statistic, *df* degrees of freedom, *Φ* phi coefficient for 2 × 2 tables, *V* Cramer’s V for larger contingency tables.

Other factors such as asthma (11.1% vs. 2.0%), nasal allergies (36.1% vs. 24.5%), frequent colds (33.3% vs. 22.4%), and acid reflux or heartburn (36.1% vs. 32.7%) were more common in the career VP group than the no VP group, but these differences did not reach statistical significance (*p* > .05). Similarly, there were no significant group differences in the presence of neurological disorders, difficulty hearing normal conversations, or regular use of medications for any condition.

In summary, the findings pertaining to health-related factors indicate that certain upper respiratory and ENT-related health issues, particularly sinus problems and other ENT disorders, may contribute to an increased risk of developing voice problems in tour guides.

### Risk factors associated with the presence of voice disorders

The association between career voice problems (VPs) and influencing variables was assessed using both unadjusted and adjusted odds ratios with 95% confidence intervals, based on the Wald forward selection criteria. Variables that were significantly associated with the presence of VPs among tour guides are presented in Table 7.

**Table 7:** Factors associated with the presence of voice problems in tour guides

Factors	Unadjusted Odds Ratio (95% CI)	*p*	Adjusted Odds Ratio (95% CI)	*p*
Speaking loudly	7.875 (0.950–65.294)	0.056	2.607 (0.235–28.941)	0.435
Speaking while having a voice problem	5.952 (1.985–17.848)	0.001*	3.476 (0.972–12.431)	0.055
Noisy group	2.481 (1.026–5.998)	0.044*	1.608 (0.555–4.658)	0.382
Guiding duration per tour	3.006 (0.983–9.190)	0.074	2.482 (0.676–9.117)	0.171
Sinus problem	3.900 (1.503–10.121)	0.005*	1.779 (0.572–5.539)	0.320
Other ear, nose, and throat disorders	7.667 (1.972–29.811)	0.003*	6.122 (1.331–28.167)	0.020*

* p < .05 was considered statistically significant. Abbreviations: *OR* odds ratio, *aOR* adjusted odds ratio, *CI* confidence interval, *ENT* ear–nose–throat.

In the unadjusted logistic regression analysis, the risk of reporting VPs was sixfold higher in guides who reported speaking while having a voice problem (OR = 5.952, 95% CI = 1.985–17.848, *p* = .001), 2.5-fold higher in those who conducted tours for noisy groups (OR = 2.481, 95% CI = 1.026–5.998, *p* = .044), 3.9-fold higher in those with sinus problems (OR = 3.900, 95% CI = 1.503–10.121, *p* = .005), and 7.7-fold higher in those with other ENT disorders (OR = 7.667, 95% CI = 1.972–29.811, *p* = .003). Although speaking loudly (OR = 7.875, 95% CI = 0.950–65.294, *p* = .056) and guiding duration per tour (OR = 3.006, 95% CI = 0.983–9.190, *p* = .074) approached significance, they were not statistically significant in the unadjusted model.

Multivariable logistic regression analysis revealed that the risk of developing VPs was sixfold higher in tour guides with other ENT disorders (adjusted OR = 6.122, 95% CI = 1.331–28.167, *p* = .020), independent of other variables. Speaking while having a voice problem also approached statistical significance in the adjusted model, with guides being 3.5-fold more likely to report VPs (adjusted OR = 3.476, 95% CI = 0.972–12.431, *p* = .055). Other variables, including guiding noisy group, sinus problems, guiding duration per tour, and speaking loudly, did not retain significance in the adjusted model.

## Discussion

This study examined the frequency, symptoms, and associated risk factors of voice problems among tour guides. The absence of significant differences between the groups in terms of age, gender, and educational level (Table 1) may indicate that the risk profile is more closely associated with other factors (e.g., occupational or health-related) than with demographic characteristics. Accordingly, the Discussion is organized under the following headings: prevalence, symptoms, phonotraumatic behaviors, occupational factors, lifestyle, and health-related factors.

### Prevalence

In this study, a high prevalence of self-reported voice problems was identified among tour guides. At the time of the questionnaire (point prevalence), 16.5% of participants reported a current voice problem, while 42.4% indicated having experienced a voice problem at some point during their professional careers. This proportion is higher than the prevalence reported in the general population (6.2%) [6]. These findings are consistent with previous studies demonstrating that voice disorders are more common among tour guides than in the general population [9]. The prevalence detected in the present study appears comparable to that reported in French tourist guides in terms of point prevalence (21%) and the proportion of guides complaining of episodes of voice disorders (45%) [11]. Similarly, a Finnish study found that 11% of guides reported four or more recurrent vocal symptoms, meeting the criteria for a voice disorder [9]. As suggested by Cansu (2020), tourism volume may influence the prevalence of voice disorders [9]. According to the Directorate of Communications, approximately 4.5 million visitors traveled to Cappadocia in 2024, indicating substantial tourist density for a relatively small region [21]. Therefore, differences across settings may be related, at least in part, to tourism volume and the frequency and intensity of guiding.

Previous studies have reported that the rates of experiencing voice problems are markedly high among intensive voice users, such as teachers and singers (57.7% and 46.09%, respectively) [6, 22]. Considering that tour guides also represent a professional group that relies on their voice as a primary tool of communication [9], and given the prevalence rate identified in the present study (42.4%), these findings suggest that tour guides may likewise be at considerable risk of developing voice disorders.

### Symptoms

Various vocal symptoms have been reported among professionals in occupations with high vocal demands. For example, a meta-analysis reported that the most common (46%) symptom among university faculty members was throat dryness [23]. Among call center workers, throat dryness and voice breaks were among the most frequently reported symptoms, and most participants described their voice as hoarse or strained [24].

In the present study, the majority of tour guides reported multiple symptoms such as throat dryness (77.6%), vocal fatigue (65.9%), throat tightness (62.4%), throat discomfort (62.4%), and hoarseness (56.5%); these rates were particularly higher in the career VP group (Table 2). Similarly, in Finland, the most frequently reported symptoms among tour guides were throat clearing, and hoarse or strained voice [9]. Sanssené et al. (2019) reported that 44.9% of tour guides experienced aphonia at least once a year, while Ulusoy Mutlu et al. (2024) identified hoarseness as the most common occupational problem among guides (15.6%) [11, 15].

In this study, all voice-related symptoms except shortness of breath were significantly more frequent in the career VP group than in the no VP group (Table 2). This finding indicates that the symptom burden was higher in the career VP group. This finding also suggests that voice problems were not limited to a single symptom but instead manifested as multiple concurrent symptoms. Therefore, regular monitoring and education of tour guides regarding vocal health may be valuable topics for future occupational health initiatives and research.

### Phonotraumatic behaviors

According to the present findings, phonotraumatic behaviors were common among tour guides; in particular, the participants frequently reported speaking loudly, excessive talking, and speaking while having a voice problem (Table 3). Similarly, in a previous study, the vast majority (92.3%) of museum and tour guides in Finland reported increasing their vocal loudness while guiding [9]. Such behaviors have been associated with increased vocal load and adverse vocal outcomes and may contribute to the development or persistence of voice problems [25]. Therefore, occupational voice use in tour guides warrants further attention, and preventive approaches such as voice hygiene education and the use of voice amplification devices may be considered for future evaluation in this professional group.

We found that the career VP group and the no VP group differed significantly in speaking loudly and speaking while having a voice problem (Table 3). This finding suggests that these two behaviors may represent primary phonotraumatic factors. In line with this finding, it has been reported that these behaviors are common among priests and that they do not receive adequate vocal rest [26]. Continuing to speak despite symptoms may increase the phonotraumatic load among tour guides; indeed, this variable approached statistical significance in the multivariable analysis (Table 7). Moreover, similar tendencies observed among French tour guides have been associated with seasonal and insecure working conditions [11]. This finding suggests that the tendency toward phonotraumatic behaviors may also be related to economic factors. Therefore, these findings raise hypotheses that occupational and economic factors may influence vocal behaviors, and may inform future policy discussions regarding professional sustainability.

Holding breath while speaking was more common in the career VP group than the no VP group and approached statistical significance (Table 3). This finding suggests that training programs addressing respiratory parameters may be clinically beneficial for tour guides. Furthermore, it has been reported that most guides are unaware of how to protect their voice and are not informed about the possibility of seeking support from healthcare professionals [11]. Therefore, the observed phonotraumatic behaviors may also stem from a lack of knowledge. Thus, educational and awareness-raising initiatives could be considered and evaluated as part of future occupational health research and program development.

### Occupational factors

Tour guides frequently provide long and intensive explanations during tours. A previous study reported that speaking loudly and for extended periods to large groups was associated with hoarseness [15]. In the present study, voice use per week exceeding 21 h was more frequent in the career VP group; however, this difference did not reach statistical significance (Table 4). It would therefore be advisable to test this tendency in larger samples or through objective measurements. Indeed, Sanssené et al. (2020) reported that speaking for more than 31 h per week constituted a significant risk factor for vocal health among tour guides [11].

Working in noisy environments has consistently been associated with voice problems [1, 9, 11]. For tour guides, raising their voice is often inevitable, particularly when speaking to large groups or under poor acoustic conditions. In the present study, group noisiness and long tour durations differed significantly between the groups (Table 4). Similarly, among French guides, noise was also reported to have an adverse association with voice quality [11]. Although Cansu (2020) did not find a significant association with vocal symptoms, background noise was among the most commonly reported environmental risk factors [9]. In the current study, although 85% of participants reported background noise, this variable did not reach statistical significance (Table 4); suggesting that the perceived noisiness of the group itself, rather than ambient noise alone, may be more closely associated with self-reported vocal load.

Although not significant, the proportion of guides exposed to poor acoustics environment, duration of high season in months, and dry or dusty weather is higher in the career VP group (Table 4). In the present study, most guides worked outdoors, in valleys, underground cities, and historical sites, settings that have been described as physically unfavorable working conditions [27]. Moreover, conducting tours in areas characterized by temperature changes and relatively dusty and dry air may further increase vocal load. The impact of such environmental factors on vocal health has been emphasized in the literature [1, 9]. However, since these variables did not reach statistical significance in the current study (Table 4), further research is warranted.

Ergonomic measures such as the use of amplification devices may help protect the vocal health of tour guides; however, most guides do not use these devices, as they feel such tools are unnecessary [9]. In the present study, it was also observed that the use of voice amplification systems was rare. Encouraging the use of amplification systems to reduce vocal load and implementing awareness programs to increase user acceptance are important [28]. In addition, incorporating regular rest breaks during tours and organizing smaller tour groups may be worth exploring as possible ways to reduce vocal load.

### Lifestyle and health

No significant differences were found between the groups in terms of lifestyle-related factors (Table 5). In both groups, the consumption of spicy and fatty foods was common. It is known that such dietary habits have been reported to trigger reflux [29–31]. In the context of the present study, both groups appear to carry a potential risk. In addition, the frequent consumption of very hot or cold foods/drinks, as well as irregular meal timing, was also observed. These habits may negatively affect vocal health by increasing the risk of reflux [32], as reflux can be an underlying cause of symptoms such as throat clearing, hoarseness, and dysphonia [33]. Therefore, it is evident that all these factors have the potential to adversely affect the voice.

A noteworthy trend was observed regarding participants’ sleep patterns: the proportion of those with regular sleep was 58.3% in the career VP group and 75.5% in the no VP group (Table 5). Considering that sleep duration has been associated with dysphonia [34] and sleep quality with voice quality [35], it can be suggested that having irregular sleep patterns may increase the risk of voice disorders among participants. This tendency indicates the need for further investigation over the long term.

High caffeine consumption and inadequate hydration were observed in both groups (Table 5). It is well established that dehydration and caffeine intake adversely affect vocal fold physiology, and that adequate hydration is essential for optimal vocal function [31, 36, 37]. Within the scope of the present study, it can be stated that hydration was not maintained at an optimal level. Although no statistically significant difference was found, the overall lifestyle patterns observed in both groups present potential risks in terms of vocal hygiene. In a study involving tour guides, medical recommendations included resting the voice (20%) and increasing water intake (6.7%) [15], suggesting that preventive approaches have a certain degree of recognition in clinical practice. On the other hand, daily physical activity was found to be high in both groups (Table 5), which may contribute positively to vocal control and breathing [38, 39].

According to the health-related findings, sinus problems were significantly more prevalent in the career VP group (Table 6). Chronic sinusitis is recognized as a biological factor that can co-occur with or contribute to voice symptoms, and it has been reported that the severity of sinusitis is associated with vocal function [40, 41]. In addition, sinus infections and other sinonasal factors may be accompanied by laryngitis or laryngeal edema [42, 43]. The chronic form of laryngitis may also result in symptoms such as hoarseness [44]. Although not statistically significant, the more frequent colds observed in the career VP group may reflect this relationship reported in the literature. It has been noted that exposure to cold weather and low humidity conditions can increase the incidence of respiratory infections [45]. Guiding in outdoor environments may therefore coincide with more frequent respiratory symptoms, which could plausibly be related to voice complaints; however, the directionality of these associations cannot be established in the present cross-sectional design. Nasal allergies and asthma were also more common in the career VP group, though not at a statistically significant level; these conditions have been associated with vocal symptoms in prior work [9, 46]. Allergies can also alter resonance through increased secretion and airway edema [47], which may help explain persistent symptoms in some tour guides.

Approximately one-third of the guides in both groups reported experiencing acid reflux or heartburn; however, these complaints did not differ significantly between the groups (Table 6). To determine the true prevalence of reflux, the use of objective tests such as pH-impedance monitoring or validated scales may be recommended. Although the difference was not statistically significant, reflux is a well-known risk factor for voice disorders [48]; therefore, these rates are clinically noteworthy.

Overall, sinus and ENT-related conditions were prominently associated with self-reported voice problems in our sample. The multivariable analysis (Table 7) is noteworthy, particularly due to the statistical significance observed for other ENT disorders. These findings suggest that vocal health among tour guides is related not only to occupational conditions but also with lifestyle and general health parameters. Prior research has linked voice problems to outcomes such as reduced professional performance, job dissatisfaction, and diminished social interaction [15, 40]. Therefore, multiple parameters, including voice hygiene education, adequate hydration, balanced nutrition, and the management of respiratory conditions, may be important for both professional functioning and overall quality of life.

In this study, some factors were statistically significant, while others were noteworthy at the trend level. Even factors that did not reach statistical significance may still contribute cumulatively to voice complaints and should not be overlooked. The literature also supports that vocal health is determined by the interaction of multiple factors [40]. Although statistical significance was not reached, more than half of the tour guides reporting voice problems had over ten years of professional experience (Table 1), suggesting possible long-term effects of cumulative vocal load. This tendency indicates that professional seniority should be examined as a potential correlate in studies with larger samples. In light of the findings, tour guiding appears to be a profession with substantial vocal demands and a notable burden of self-reported voice problems.

Interpretation of the observed associations should also consider alternative explanations and potential confounding. For example, workload characteristics that were not fully captured (e.g., variability in tour intensity across seasons, venue type, group size, and use of amplification), health status (e.g., reflux, allergies/asthma, medication use), and lifestyle factors (e.g., hydration patterns, caffeine intake, sleep, and smoking) may influence both exposure variables and voice complaints. Because outcomes and exposures were assessed concurrently and relied on self-report, reverse causation (e.g., individuals with voice problems being more likely to perceive or report certain risk factors) and shared-method bias are possible, and residual confounding cannot be excluded. Accordingly, the findings should be interpreted as associations rather than evidence of direct effects.

Although the findings provide valuable insights into the vocal health of tour guides, several limitations should be acknowledged. First, this study used a cross-sectional, survey-based design with voluntary response recruitment from a single regional professional chamber (the Nevşehir Chamber of Tour Guides; NERO) in Cappadocia; therefore, selection and non-response bias cannot be ruled out, and the results may not be generalizable to tour guides working in other regions, countries, or tourism settings with different workloads, languages, or acoustic/environmental conditions. Second, the modest sample size (*n* = 85) and response rate (18.0%) may have limited statistical power to detect small-to-moderate associations and may have contributed to wide confidence intervals in regression analyses. Additionally, the limited number of outcome events may constrain model stability. Third, outcomes and exposures were self-reported and may be affected by recall and reporting biases. Fourth, the questionnaire was researcher-developed and, although face and content validity were supported through expert review, formal psychometric evaluation (e.g., construct validity testing and test–retest reliability) was not performed. The cross-sectional design also limits causal interpretation. Finally, no objective clinical assessment (e.g., laryngoscopic and/or stroboscopic evaluation) was conducted; therefore, the presence and type of vocal fold pathology among participants reporting voice problems could not be confirmed, and objective acoustic/aerodynamic measures were not obtained. Future research incorporating laryngeal visualization alongside objective voice measures, preferably using longitudinal designs and multi-site sampling across different tourism contexts, would provide a more comprehensive understanding of occupational voice risks in this population.

Despite these limitations, our findings suggest that tour guides may be an important population for future occupational voice health research and needs assessments. Preventive approaches such as voice hygiene education, opportunities for vocal rest, and ergonomic or acoustic considerations (e.g., amplification in noisy venues) may warrant evaluation in larger, multi-site, and preferably longitudinal studies. In this context, screening-oriented components should be considered as hypotheses for further investigation rather than immediate implementation recommendations. Collaborative work between speech-language pathologists, occupational health stakeholders, and tourism organizations may help inform the design and feasibility of such interventions.

From an occupational health perspective, tourism organizations and professional bodies may consider exploring practical measures that could reduce vocal load, pending feasibility evaluation in broader samples. These may include structured voice-care training delivered by speech-language pathologists (voice hygiene and efficient projection), wider availability and acceptability of portable amplification in noisy venues, and facilitating short vocal rest opportunities during tours. Ensuring access to water may support hydration practices. Establishing referral pathways for ENT evaluation may also be relevant for guides with persistent symptoms. Importantly, these suggestions should be interpreted as considerations for future program development and evaluation rather than prescriptive implementation guidance based on the present cross-sectional data.

## Conclusion

Voice problems were common among professional tour guides in Cappadocia, with throat dryness, vocal fatigue, and hoarseness being the most frequently reported symptoms. Self-reported voice problems were associated with phonotraumatic behaviors, challenging working conditions, and certain health-related factors, particularly ENT disorders. In the multivariable model, ENT disorders remained an independent predictor, whereas speaking while symptomatic showed borderline significance. These findings highlight the need for further research on occupational voice health in tour guides and may help inform future preventive efforts.

## Supplementary Information

Below is the link to the electronic supplementary material.

Supplementary Material 1 (DOCX 25.2 KB)

## Data Availability

The data that support the findings of this study are available from the corresponding author upon reasonable request.
