# Supplementary material for: Prevalence and risk factors of voice disorders among tour guides in Cappadocia
Source: Eur Arch Otorhinolaryngol. 2026 May 28;283(7):4553–68. doi: 10.1007/s00405-026-10328-3 (PMC13388353; doi:10.1007/s00405-026-10328-3)
Supplement: Supplementary file 1 — Supplementary Material 1 (DOCX 25.2 KB) [file 405_2026_10328_MOESM1_ESM.docx]

**Questionnaire on the Prevalence of Voice Disorders and**

**Related Risk Factors Among Tour Guides**

Please read the following questions carefully and select the most appropriate answer.

**Demographic Information**

1) Age: …….. years

2) Gender:

 Female  Male

3) Education level:

 Undergraduate  Graduate

4) How many years of professional experience do you have as a tour guide?

 0–4 years  5–10 years  More than 10 years

5) Do you have another job that requires prolonged voice use?

 Yes  No

6) Do you have a voice-related hobby (e.g., singing, public speaking, streaming)?

 Yes  No

**Prevalence of Voice Problems**

1) Are you currently experiencing any voice problems?

 Yes  No

2) Have you ever experienced voice problems at any time during your professional career as a tour guide?

 Yes  No

3) Have you ever experienced complete voice loss (aphonia) during your professional activities as a tour guide?

 Never  Once or twice per year  Three or more times per year

**Symptoms Related to Voice Problems**

1) Hoarseness:

 Yes  No

2) Throat dryness:

 Yes  No

3) Vocal fatigue:

 Yes  No

4) Throat tightness:

 Yes  No

5) Sudden loss of voice:

 Yes  No

6) Throat discomfort:

 Yes  No

7) Shortness of breath:

 Yes  No

8) Difficulty speaking loudly:

 Yes  No

9) Throat pain:

 Yes  No

10) Complete voice loss:

 Yes  No

**Phonotraumatic Behaviors**

1) Speaking loudly:

 Yes  No

2) Excessive talking:

 Yes  No

3) Speaking at an excessively fast rate:

 Yes  No

4) Frequent coughing:

 Yes  No

5)Frequent throat clearing:

 Yes  No

6) Holding breath while speaking:

 Yes  No

7) Speaking during a throat infection:

 Yes  No

8) Speaking while having a voice problem (e.g., hoarseness):

 Yes  No

**Occupational Risk Factors Related to Tour Guiding**

1) What is the average group size you guide during the high season?

 1–10 people  11–30 people  31 or more people

2) In which settings do you need to speak loudly? (You may select more than one option)

 Indoor settings  Outdoor settings

3) What factors do you think cause you to speak loudly in these settings? (You may select more than one option)

 Poor acoustics of the environment

 Presence of background noise

 The group being dispersed

 The group being noisy

4) During tour guiding, how many hours per week do you use your voice?

 2–10 hours  11–20 hours  More than 21 hours

5) During tour guiding, how many hours per week do you speak at a high volume?

 Less than 5 hours  6–15 hours  16–20 hours  More than 21 hours

6) During a single tour, how long do you usually provide guiding on average?

 5 hours or less  6 hours or more

7) In a typical year, for how many months is it high season?

 1–6 months  7–12 months

8) Do you use any voice amplification devices (e.g., megaphone) during tours?

 Never  Rarely  Sometimes  Often  Always

9) In which types of locations do you usually provide tour guiding? (You may select more than one option)

 Museums (indoor)

 Museums (outdoor)

 Underground cities and cellars

 Valleys

 Fairy chimneys

 Tour buses

 City tours

 Historical buildings and structures

 Churches and mosques

10) Do temperature changes occur during your tours?

 Never  Rarely  Sometimes  Often  Always

11) Is the weather windy during your tours?

 Never  Rarely  Sometimes  Often  Always

12) Is the weather humid during your tours?

 Never  Rarely  Sometimes  Often  Always

13) Is the weather dusty during your tours?

 Never  Rarely  Sometimes  Often  Always

14) Is the weather dry during your tours?

 Never  Rarely  Sometimes  Often  Always

15) Do you experience echo during your tours?

 Never  Rarely  Sometimes  Often  Always

16) Do you guide in environments with disturbing background noise during your tours?

 Never  Rarely  Sometimes  Often  Always

17) Do you experience stress and anxiety while performing your tour guiding profession?

 Never  Rarely  Sometimes  Often  Always

**Lifestyle-Related Risk Factors**

1) Do you consume spicy or fatty foods?

 Yes  No

2) Do you consume very hot or very cold foods/drinks?

 Yes  No

3) Do you have irregular eating habits? (e.g., eating late at night)

 Yes  No

4) Do you smoke or use nicotine products?

 Yes  No

5) Do you have a regular sleep routine?

 Yes  No

6) How many hours do you usually sleep per night?: ……..

7) Daily amount of beverage consumption

1. Coffee:

 2 cups or less  More than 2 cups

1. Energy drink/Cola:

 2 cups or less  More than 2 cups

1. Tea:

 2 cups or less  More than 2 cups

1. Water:

 8 cups or less  More than 8 cups

1. Alcohol:

 None  2 cups or less  More than 2 cups

8) Daily physical activity:

 Less than 30 minutes  More than 30 minutes

**Health-Related Risk Factors**

1) Asthma:

 Yes  No

2) Sinus problems (e.g., sinusitis):

 Yes  No

3) Nasal allergies (e.g., hay fever, allergic rhinitis):

 Yes  No

4) Frequent colds:

 Yes  No

5) Difficulty in hearing normal conversations:

 Yes  No

6) Acid reflux or heartburn:

 Yes  No

7) Neurological disorders:

 Yes  No

8) Regular medication use:

 Yes  No

9) Other ear, nose, and throat disorders:

 Yes  No
